# Tube Feeding-Related Bowel Ischemia Presenting As Extensive Intestinal Pneumatosis Complicated With Hepatic Portal Venous Gas

**DOI:** 10.7759/cureus.24313

**Published:** 2022-04-20

**Authors:** Eunice Amenu, Sahar Karim, Rafael C Da Silva

**Affiliations:** 1 Internal Medicine, Piedmont Athens Regional Medical Center, Athens, USA; 2 School of Medicine, Universidade Para o Desenvolvimento do Alto Vale do Itajaí, Rio do Sul, BRA

**Keywords:** portal vein, mesenteric ischemia, mortality, treatment complication, percutaneous endoscopic gastrojejunostomy, enteral feeding, nonocclusive bowel ischemia, pneumatosis intestinalis, hepatic portal venous gas

## Abstract

Hepatic portal venous gas (HPVG) is a condition where air embolization from disrupted intestinal mucosa reaches the portal system. It is an uncommon finding and denotes severity. This report describes HPVG as a rare and lethal complication of enteral nutrition. The patient had a history of pancreatic adenocarcinoma (PADC) managed with Whipple’s surgery, complicated with poor oral intake, requiring percutaneous jejunostomy. Subsequently, the patient presented with severe abdominal pain and distension. On imaging, he was found to have diffuse pneumatosis intestinalis (PI) and HPVG. The patient underwent exploratory laparotomy with intraoperative findings of bowel ischemia starting at the distal point of the enteral tube feeding. Despite aggressive intensive support, the patient died. The aim of this case description is to highlight a rare complication of enteral feeding with impressive imaging findings. Intensive care providers should consider this complication in patients with acute abdominal symptoms, who are under enteral feeding.

## Introduction

Hepatic portal venous gas (HPVG) is an uncommon imaging finding and is usually related to pneumatosis intestinal (PI) secondary to bowel ischemia. Unfortunately, this clinical condition is associated with poor prognosis and high mortality. Enteral nutrition through tube feedings is a common medical procedure for patients with gastrointestinal conditions and the inability to oral feeding. The aim of this report is to present a rare complication of enteral nutrition leading to bowel ischemia, pneumatosis intestinal, and hepatic portal venous gas after Whipple’s procedure for pancreatic cancer.

## Case presentation

A 64-years-old male with a history of pancreatic adenocarcinoma (PDAC) presented to the hospital with worsening abdominal pain, distension, nausea, and stopping of intestinal transit. The patient's PDAC was categorized as Stage IA (American Joint Committee on Cancer), and further subcategorized as a resectable disease. Neoadjuvant therapy was not performed. He underwent Whipple’s surgery four months ago, complicated with poor oral intake. A percutaneous jejunostomy was placed two months later for distal enteral feeding. Chemotherapy was started at the same time including gemcitabine plus capecitabine. Upon hospital arrival, a physical exam revealed a chronically ill-appearing elderly adult with BP of 126/84 mmHg, a pulse of 133/bpm, a temperature of 97.2 ˚F, and a respiratory rate of 24/ipm. The abdomen was firm, markedly distended, and tender to palpation. The midline incision was well-healed. Percutaneous jejunostomy was in place and upon gravity bag drainage, immediate efflux of a large amount of bloody drainage and gas was noticed. The initial comprehensive metabolic panel is shown in Table 1.

**Table 1:** Comprehensive metabolic panel upon admission.

Laboratory (normal range)	Results
White blood cells (4.5-11.0 x 10^3^/mm^3^)	7.0 10^3^/mm^3^
Hemoglobin (13.0-18.0 g/dL)	15.8 g/dL
Platelets (130-400 x 10^3^/µL)	318 10^3^ /µL
Bands (0-5%)	40%
Sodium (135-145 mmol/L)	126 mmol/L
Potassium (3.4-5.0 mmol/L)	3.6 mmol/L
Chloride (95-108 mmol/L)	88 mmol/L
Bicarbonate (20-32 mmol/L)	20 mmol/L
BUN (8-25 mg/dL)	15 mg/dL
Creatinine (0.7 to 1.3 mg/dL)	1.09 mg/dL
ALT (10-55 units/L)	> 5000 U/L
AST (10-40 units/L)	> 10000 U/L
Bilirubin, total (0.0-1.0 mg/dL)	0.9 mg/dL
Albumin (3.1 – 4.3 g/dL)	3.8 g/dL
Lactic acid (0.5 to 2.2 mmol/L]	5 mmol/L
Anion Gap (8-12)	20
Blood and urine cultures	Negatives

**Figure 1:**
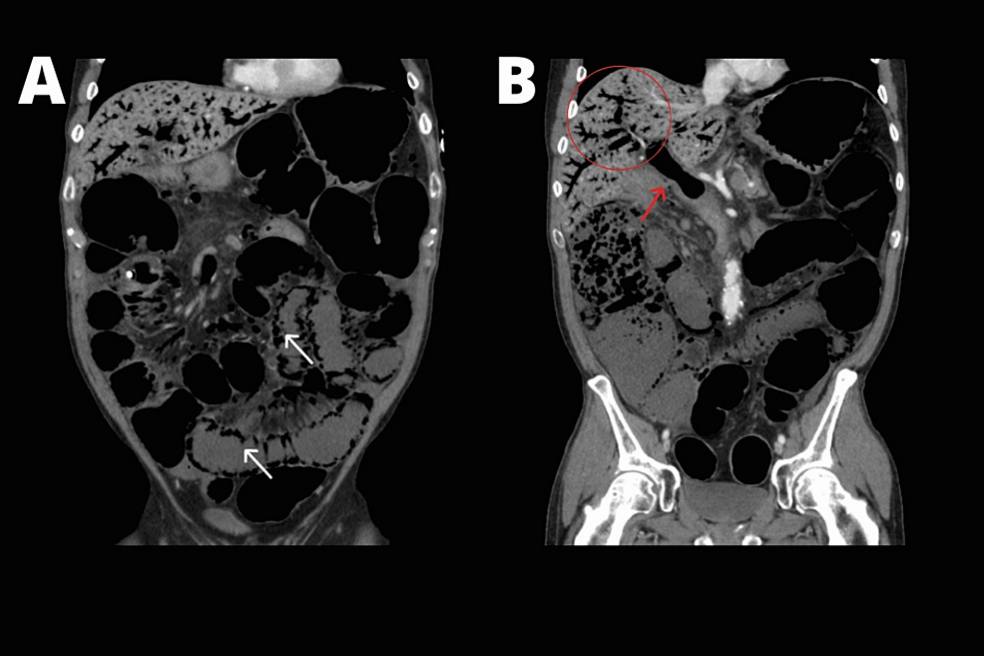
Contrast-enhanced computerized tomography of the abdomen. In A, extensive pneumatosis instestinalis (white arrows). In B, gas is seen inside the portal vein (red arrow) along with extensive intra-hepatic portal venous gas (red circle).

He was admitted into the intensive care unit (ICU) on account of rapid deterioration of vital signs. Broad-spectrum antibiotics were started along with fluid resuscitation and vasopressor drip. Contrast-enhanced computerized tomography (CT) of the abdomen was obtained (Figure 1). 

Surgery was consulted and the patient underwent exploratory laparotomy with intraoperative findings of grossly ischemic liver and small bowel and splenic flexure ischemia. Partial colectomy, small bowel resection, and partial hepatectomy were performed. His hospital course was complicated by persistent septic shock and anuric acute kidney injury requiring continuous renal replacement therapy. After a protracted postoperative course, he continued to deteriorate, and given his poor premorbid state and prognosis, the decision was made for palliation. Histology of the resected bowel showed mucosal necrosis and submucosal vascular thrombosis compatible with ischemia. Liver tissue was consistent with diffuse general coagulative necrosis. 

## Discussion

The patient's presentation is remarkable for a rapid deterioration of his clinic condition in the setting of non-occlusive bowel ischemia (NOBI) leading to pneumatosis intestinalis (PI) and ultimately hepatic portal venous gas with liver ischemia. Before presentation, the patient was on percutaneous jejunostomy tube-feeding with the distal tip of the catheter at the same point where the small bowel ischemia was noticed. There was no history of previous persistent hypotension. In addition, the patient underwent chemotherapy (gemcitabine and capecitabine), which could negatively impact the intestinal mucosa integrity. Feeding-related bowel ischemia or nonocclusive bowel ischemia (NOBI) associated with early enteral nutrition (EN) presents with nonspecific clinical features [1]. Early signs and symptoms include crampy abdominal pain and bloating. The late presentation includes abdominal distension and paralytic ileus. Transmural bowel necrosis may also develop; with progression to septic shock and organ failure [1]. Most of the cases of feeding-related bowel ischemia reported in literature showed that it occurred after patients had been enterally fed for an average of eight days [2,3]. Our patient had a jejunostomy done two months before presentation. It was later than comparative literature. Radiological findings include PI (88%), free fluid (38%), thickened/dilated bowel loops (38%), or free peritoneal air (25%) [2]. Our patient presented with diffuse PI both on small and large bowels. There are several theories of how EN contribute to NOBI, three of which are outlined below. Obligatory absorption of intraluminal nutrients increase energy demands in metabolically stressed enterocytes [2]. EN administered in the setting of ileus allows bacterial overgrowth causing progressive distention, which impairs mucosal perfusion [2]. A third theory is that EN generates intraluminal toxins that cause direct mucosal injury [2]. Unfortunately, our patient presentation of bowel ischemia and diffuse PI complicated with diffuse hepatic portal venous gas, which led to liver ischemia and shock liver. 

HPVG, a rare radiological finding, is defined as a branching radiolucency on x-ray or computorized tomography scan within 2 cm beneath the liver capsule [4]. HPVG often has a relatively short duration and is not an independent disease [5]. It is a critical condition with high mortality. A 2001 review of 182 cases showed an overall mortality as high as 39%, with only 3% of HPVG cases associated with intraperitoneal tumor [6]. The presence of HPVG with pneumatosis intestinalis (PI) indicates a high probability of bowel gangrene [7] and high mortality; 49% mortality with PI and 29% without PI [8]. Although mechanism is not well understood, it is associated with bowel distension, elevation of digestive tract pressure, endoscopic procedures, mucosal damage, sepsis, and gas embolization among others [4, 9, 10].

Treatment for HPVG should be based on the underlying cause of disease and involves conservative or surgical management [5]. Surgery is indicated in portal-mesenteric vein gas and the presence of bowel ischemia [11]. Stopping tube feeding is paramount, and early parenteral nutrition should be considered. Our patient showed small bowel ischemia just distal to the tip of his feeding tube. This likely led to microcirculation impairment, organ hypoperfusion and additional ischemia at watershed areas on the splenic flexure, explaining the involvement of the large bowels. Ultimately, with the progression of mucosa injury and diffuse pneumatosis intestinalis, patient developed extensive hepatic portal venous gas and subsequent ischemic liver injury. 

## Conclusions

Hepatic portal venous gas is a high-risk condition associated with elevated mortality. Rarely, it is associated with enteral nutrition. Increased energy demand in metabolically stressed enterocytes, bacterial overgrowth causing progressive distention, impaired mucosal perfusion, and intraluminal toxins causing direct mucosal injury can explain the development of this condition. The treatment depends upon clinical presentation. The base of therapy should focus on stopping tube feedings, early parenteral nutrition, and surgery in the setting of bowel ischemia.
